# Antipsychotic Treatment and Longitudinal Body Mass Index Trajectories in Youth with and Without Autism Spectrum Disorder

**DOI:** 10.3390/jcm15020508

**Published:** 2026-01-08

**Authors:** Javier Sánchez-Cerezo, Rocío Paricio Del Castillo, Lourdes García-Murillo, Gustavo Centeno-Soto, Mónica Jodar Gómez, Belén Ruiz-Antorán, Inmaculada Palanca-Maresca

**Affiliations:** 1 Department of Psychiatry, Division of Child and Adolescent Psychiatry, Puerta de Hierro University Hospital, Majadahonda, 28222 Madrid, Spain; 2 Department of Clinical Pharmacology, Puerta de Hierro University Hospital, Majadahonda, 28222 Madrid, Spain

**Keywords:** autism spectrum disorder, antipsychotics, BMI, weight gain

## Abstract

**Background:** Children and adolescents with autism spectrum disorder (ASD) frequently receive antipsychotics and are considered at increased risk for weight gain. Few studies have compared longitudinal weight trajectories between youth with ASD and those with other psychiatric disorders. **Methods:** This naturalistic, registry-based study used data from the SENTIA cohort, which prospectively monitors antipsychotic safety in individuals under 18 years at a university hospital in Spain. Clinical characteristics were compared between participants with and without ASD. Longitudinal body mass index (BMI) z-score trajectories were analysed using linear mixed-effects models. **Results:** The sample included 266 participants, of whom 113 (42.5%) had ASD. Individuals with ASD were more often male and initiated antipsychotic treatment at a younger age. Of the 26 participants prescribed an antipsychotic before age 6, 88.5% had ASD. Comorbidity profiles were similar across groups. Risperidone and aripiprazole were the most frequently prescribed antipsychotics. BMI z-scores increased over time (β = 0.130, *p* = 0.017), and baseline BMI z-score was the strongest predictor. ASD diagnosis did not modify the average linear rate of BMI z-score change (time × ASD: *p* = 0.251); however, a significant quadratic time × ASD interaction (β = −0.016, *p* = 0.041) was consistent with a more pronounced early increase followed by earlier attenuation of BMI z-scores in the ASD group. **Conclusions:** Although antipsychotic treatment was initiated earlier in youth with ASD, no clear difference was observed in the rate of BMI z-score change. Differences in weight trajectories underscore the need for metabolic monitoring in antipsychotic-treated youth.

## 1. Introduction

Autism spectrum disorder (ASD) is an early-onset neurodevelopmental condition characterised by persistent deficits in social communication and interaction, alongside restricted or repetitive behaviours [1]. Global autism prevalence varies across regions, with a median prevalence of 100 per 10,000 [2]. The median male-to-female ratio is 4.2 [2], although it may be lower [3].

ASD is frequently accompanied by psychiatric and somatic comorbidities. Disruptive behaviours such as hyperactivity, irritability, and self-harm are common [4]. Individuals with ASD have also been reported to be particularly vulnerable to weight gain and metabolic disorders [5]. Obesity is more prevalent in children and adolescents with ASD than in their peers without ASD [6,7]. Consistently, a longitudinal study reported that individuals with ASD also show a higher prevalence of metabolic syndrome, with reported rates of 31.5% for dyslipidaemia, and 19.4% for hypertension and type 2 diabetes [8].

A range of pharmacological interventions has been evaluated for behavioural symptoms in ASD. However, only two second-generation (atypical) antipsychotics—aripiprazole and risperidone—have received approval from the Food and Drug Administration (FDA) for irritability associated with ASD in children and adolescents in the United States of America (US) [9,10]. In Europe, the European Medicines Agency (EMA) has approved haloperidol for persistent severe aggression in children and adolescents with ASD [11]. Consequently, antipsychotic prescribing in this population often occurs in off-label contexts. Off-label use in children and adolescents may involve unlicensed indications, doses, or durations exceeding the product information, and use outside the specified age range, potentially affecting efficacy and tolerability [12].

Antipsychotic prescribing in children and adolescents has increased worldwide [13]. Prescription rates vary by country [13], with the highest prevalence estimates (~3% among those aged ≤19 years) reported in Taiwan and the US [14]. Off-label prescribing rates range from 36 to 93.2%, with increases over time in both incidence and prolonged use [15,16,17]. It has been estimated that 16.6% of children and adolescents with ASD are prescribed antipsychotic medications [18]. Although second-generation antipsychotics (SGA) are often preferred because they cause fewer extrapyramidal symptoms, they have a greater propensity for weight gain and metabolic disturbances [19].

A retrospective study by Yoon et al. (2016), involving 202 young people with ASD, found that treatment with olanzapine, risperidone, and aripiprazole was associated with increases in BMI z-scores, with olanzapine producing the greatest gain [20]. Similarly, a study in paediatric patients with neurological disorders treated with risperidone reported that 53.8% experienced at least one metabolic side effect, most commonly hyperlipidaemia (34.6%) [21]. Neither study included a comparison group.

Taken together, children and adolescents with ASD appear to have a baseline vulnerability to weight and metabolic issues, and the literature indicates that they are at heightened risk for antipsychotic-induced weight gain [20]. Nevertheless, there is a paucity of research directly comparing longitudinal weight trajectories between children and adolescents with ASD and their peers with other psychiatric disorders. Such comparisons are essential to clarify whether ASD constitutes a distinct risk profile in the context of antipsychotic exposure. By leveraging a prospective pharmacovigilance registry with repeated longitudinal measurements, this study extends prior short-term or pre–post analyses and provides real-world evidence relevant to long-term metabolic monitoring in antipsychotic-treated youth.

Our naturalistic study aimed to compare trajectories of weight gain between children and adolescents with ASD and those with other psychiatric disorders who were prescribed an antipsychotic medication at a child and adolescent psychiatry outpatient clinic in Spain. We also compared prescription patterns between groups and examined clinical characteristics. We hypothesised that children with ASD would experience a greater body mass index (BMI) z-score increase at follow-up. We further hypothesised that children and adolescents with ASD would be prescribed an antipsychotic at a younger age.

## 2. Materials and Methods

### 2.1. Data

Data were obtained from the SENTIA (SafEty of NeurolepTics in Infancy and Adolescence) registry [22], which was created in 2011 to track adverse effects in children and adolescents (<18 years) treated with an antipsychotic. Data on cases were entered by reporting paediatricians and psychiatrists and managed using Research Electronic Data Capture (REDCap) 15.9.3, a secure, web-based software platform (Vanderbilt University, Nashville, TN, USA) [23]. The registry has collected data prospectively since January 2011. For the present study, we analysed data up to May 2025.

### 2.2. Participants

SENTIA [22] includes patients younger than 18 years of age who are currently taking or initiating treatment with any antipsychotic, either as monotherapy or in combination. There are no clinical exclusion criteria. Patients are included in the registry after assessment by a child and adolescent psychiatrist and a clinical pharmacologist. Diagnoses are provided by the treating child and adolescent psychiatrist. ASD diagnoses were established following an assessment by a child and adolescent psychiatrist, including the Autism Diagnostic Observation Schedule, second version (ADOS-2), or the Autism Diagnostic Interview-Revised (ADI-R) [24,25].

Antipsychotic-naïve status was defined as having a baseline assessment within 30 days of initiating antipsychotic treatment. Participants whose baseline visit occurred more than 30 days after antipsychotic initiation were classified as non-naïve.

### 2.3. Ethics

The study was conducted in accordance with the Declaration of Helsinki. The register was approved by the Research Ethics Committee of Hospital Universitario Puerta de Hierro (October 2010) and the Spanish Agency of Medicines and Medical Devices as a post-authorisation observational study (BRA-ANT-01). The project was registered with the European Network of Centres for Pharmacoepidemiology and Pharmacovigilance [22]. In order to be included in the registry, informed consent was obtained from parents or legal guardians and also from the patient if over 12 years old. Collected data were treated anonymously. Ethical review and approval were waived for this study due to the collected data being treated anonymously.

### 2.4. Statistics

Descriptive statistics were computed for all variables. Continuous variables are presented as means and standard deviations (SD), and categorical variables as frequencies and percentages. Baseline group differences between participants with and without ASD were examined using Welch’s *t*-tests for continuous variables and χ^2^ tests or Fisher’s exact tests for categorical variables, as appropriate based on cell counts.

Anthropometric variables (height, weight, and BMI) were standardised using age- and sex-specific z-scores derived from Spanish reference population tables [26]. Weight and height were measured during routine outpatient visits using standardised clinical procedures. BMI categories (thinness, normal weight, overweight, obesity) were defined according to the WHO cut-offs [27]. Group differences in anthropometric z-scores and BMI categories at baseline were assessed using Welch’s *t*-tests and Fisher’s exact tests, respectively.

To evaluate longitudinal changes in BMI z-scores, linear mixed-effects models were fitted to account for within-subject correlation due to repeated measurements. Models included a random intercept for each participant and, when supported by model comparison, a random slope for time. Fixed effects included ASD diagnosis, time since baseline visit (modelled as a continuous variable and centred at its mean), a quadratic term for time to allow for non-linear trajectories, baseline BMI z-score, sex, baseline age, stimulant use, antipsychotic-naïve status, and antipsychotic group at baseline (risperidone/aripiprazole vs. other antipsychotics). Time was centred to improve the interpretability of model parameters and reduce collinearity between linear and quadratic terms, and a quadratic time term was included to flexibly capture non-linear patterns of BMI change commonly observed after antipsychotic initiation. Information on the antipsychotic dose was not available in the registry. Interactions between ASD diagnosis and both the linear and quadratic time terms were included to test whether the magnitude and shape of BMI z-score trajectories differed by diagnostic group.

Time was defined as the elapsed time since the baseline visit recorded in the registry. For antipsychotic-naïve participants, this baseline visit closely approximated treatment initiation. In contrast, for non-naïve participants, baseline occurred after a variable period of prior antipsychotic exposure.

Model selection for the random-effects structure (random intercept vs. random intercept and slope) was based on likelihood ratio tests and information criteria. Model assumptions were assessed by visual inspection of residual plots to evaluate normality and homoscedasticity, as well as by examining leverage and influence diagnostics to identify potential outliers or influential observations. Linear mixed-effects models allow for unbalanced follow-up; therefore, all available BMI observations were included without imputation.

Sensitivity analyses were performed to evaluate the robustness of the main findings, including analyses restricted to antipsychotic-naïve participants, models including a time × age interaction, and models with a simplified random-effects structure.

For key interaction terms, effect estimates are reported together with 95% confidence intervals to quantify the range of plausible group differences over time. All statistical analyses were conducted in R (version 4.4.2, R Foundation for Statistical Computing, Vienna, Austria). Linear mixed-effects models were fitted using the *lme4* and *lmerTest* packages in R. A two-sided *p*-value < 0.05 was considered statistically significant.

## 3. Results

A total of 266 children and adolescents were included in our registry after being prescribed an antipsychotic from January 2011 to May 2025. The mean age of the sample was 11.1 years with a minimum age of 3 years and a maximum age of 17.9 years. Twenty-six (9.8%) patients were prescribed an antipsychotic before 6 years of age. See Table 1 for the sociodemographic and clinical characteristics.

In total, 113 (42.5%) individuals had a diagnosis of ASD. Family psychiatric and somatic histories are shown in Table 2.

We compared baseline characteristics between participants with and without ASD (See Table 3). Children and adolescents with ASD were considerably younger than those without ASD (mean age 9.8 vs. 12.0 years, *p* < 0.001) and more frequently male (87.6% vs. 68.6%, *p* < 0.001). Consistent with their younger age, individuals with ASD were substantially more likely to have been prescribed an antipsychotic before 6 years of age (20.4% vs. 2.0%, *p* < 0.001).

Psychiatric and somatic comorbidity rates did not differ significantly between groups. However, several family history variables showed group differences. A family history of ASD was markedly more common among participants with ASD (17.7%) than among those without ASD (3.3%) (*p* < 0.001). Conversely, a family history of substance use disorder was less frequently reported in the ASD group (0.9%) compared with the non-ASD group (7.2%) (*p* = 0.031). In addition, a family history of obesity was reported in 2.7% of participants with ASD and 9.8% of those without ASD (*p* = 0.041).

### 3.1. Prescribing Reasons

Information on the reason for initiating antipsychotic treatment was available for 63 participants (23.7%). Among these, 37 (58.7%) received the medication to target specific symptoms, and 26 (41.3%) for a diagnostic indication. The distribution of these prescribing reasons was similar between children and adolescents with and without ASD: 55.0% of antipsychotic prescriptions in the ASD group and 60.5% in the non-ASD group were initiated for symptomatic reasons, while 45.0% and 39.5%, respectively, were initiated for diagnostic reasons (*p* = 0.785).

Among the 37 participants who received an antipsychotic for symptomatic reasons, the most frequently targeted symptoms were poor sleep (81.1%) and aggressiveness (78.4%). Comparison by diagnostic group revealed that aggressiveness was less frequently the target symptom among individuals with ASD (54.5%) than among those without ASD (88.5%), a difference that reached statistical significance (*p* = 0.035). No other symptom domain showed significant group differences. Poor sleep was common in both groups (81.8% in ASD vs. 80.8% in non-ASD).

### 3.2. Anthropometrics

BMI category distributions are summarised in Table 4. There were no significant differences in BMI category or in z-scores for height, weight, or BMI between ASD and non-ASD groups (see Table 5).

**Figure 1:**
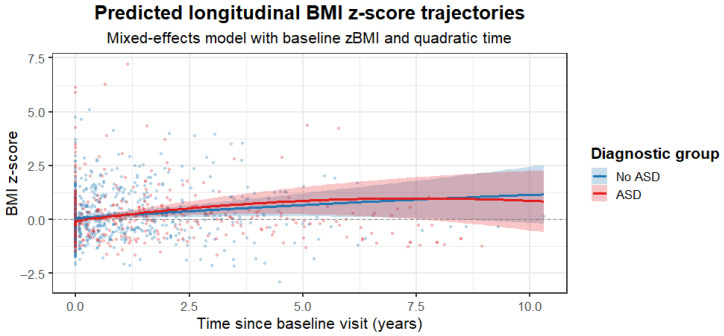
Predicted longitudinal BMI z-score trajectories.

In the final linear mixed-effects model examining longitudinal BMI z-scores, a total of 899 observations from 229 participants were included. 27 participants were excluded from the model due to missing covariate data required for model adjustment. Substantial between-subject variability was observed in baseline BMI z-scores (random intercept variance = 0.41) and in individual trajectories over time (slope variance = 0.18), with a strong correlation between random intercepts and slopes (r = 0.99). Model fit was adequate (AIC = 968.3; log-likelihood = −468.1). After adjustment for baseline BMI z-score, sex, age, stimulant use, antipsychotic-naïve status, and antipsychotic group, BMI z-scores showed a significant overall increase over time (linear time effect: β = 0.130, SE = 0.054, *p* = 0.017), indicating a modest but consistent rise in BMI across the cohort. ASD diagnosis was not associated with baseline BMI z-scores (β = 0.046, SE = 0.119, *p* = 0.700), nor with the linear rate of BMI z-score change over time (time × ASD: β = 0.097, SE = 0.084, *p* = 0.251). However, a significant quadratic interaction between time and ASD was observed (time^2^ × ASD: β = −0.016, SE = 0.008, *p* = 0.041), consistent with a more pronounced early increase followed by earlier attenuation of BMI z-score gain in the ASD group. While no statistically significant group difference was detected in the average annual slope, the 95% confidence interval for the linear time × ASD interaction (−0.07 to 0.26 BMI z-score units per year) was compatible with small to moderate clinically relevant differences and did not exclude potentially meaningful effects. None of the remaining covariates showed significant independent associations with BMI z-score trajectories. Complete model estimates are provided in Table 6, and the predicted longitudinal BMI z-score trajectories for ASD and non-ASD groups are illustrated in Figure 1.

Figure 1 footnote: Predicted BMI z-score trajectories derived from the final linear mixed-effects model adjusted for baseline BMI z-score, sex, baseline age, stimulant use, antipsychotic-naïve status, and antipsychotic group, including linear and quadratic time effects. Shaded areas represent 95% confidence intervals based on fixed effects only. The model indicates an overall increase in BMI z-scores over time, with a significant difference in the temporal shape of trajectories between the ASD and non-ASD groups.

### 3.3. Sensitivity Analyses

Given the substantial sex imbalance in the cohort, sex was included as a covariate in all adjusted models to reduce potential confounding in group comparisons. Due to the limited number of female participants, sex-stratified analyses were not performed, and results should be interpreted at the population level. Follow-up time was modelled continuously using linear and quadratic time terms within mixed-effects models, allowing for individual variability in exposure duration; however, categorical analyses based on treatment duration thresholds (e.g., <6 months vs. ≥6 months) were not feasible due to heterogeneity in follow-up and limited statistical power.

In antipsychotic-naïve participants, the quadratic time × ASD interaction remained directionally consistent with the main analysis but was estimated with reduced precision, likely due to the smaller sample size.

To examine potential developmental effects, we fitted an additional model including a time × baseline age interaction. This term was not statistically significant (*p* = 0.96), and the quadratic time × ASD interaction remained unchanged.

Finally, the model was refitted using a simpler random-effects structure (random intercept only). The main findings were robust to this simplification, with similar estimates for the time × ASD and time^2^ × ASD interactions.

## 4. Discussion

In this registry-based cohort of children and adolescents receiving antipsychotic treatment in routine clinical care, we compared clinical characteristics, prescribing patterns, and longitudinal BMI z-score trajectories between individuals with and without ASD. Consistent with previous reports, youth with ASD initiated treatment at a younger age and were more frequently male than their non-ASD peers [28]. Psychiatric and somatic comorbidity rates were otherwise broadly comparable across diagnostic groups.

Our findings differ from previous literature reporting greater vulnerability for antipsychotic-associated weight gain in children and adolescents with ASD compared with non-ASD populations [20,29,30]. These differences may be due to prior studies relying on shorter follow-up periods or pre-post designs, which primarily capture the early phase of antipsychotic-induced weight gain and may not adequately characterise its longer-term trajectory. In contrast, the present study is based on a naturalistic clinical registry with extended follow-up, greater diagnostic heterogeneity, and adjustment for baseline BMI and developmental factors. From this perspective, our findings do not exclude greater early weight gain in ASD, but suggest that such differences may attenuate over time rather than persist consistently throughout treatment.

In our sample, nearly 90% of children prescribed an antipsychotic before the age of 6 had a diagnosis of ASD, and all but one were boys. Antipsychotic prescribing in young children is uncommon. A study analysing US private insurance data reported declining antipsychotic use in children aged 2–7 years over time, with autism-related conditions representing the most frequent indication and boys being more frequently prescribed antipsychotics [28].

Risperidone and aripiprazole accounted for more than 80% of antipsychotic prescriptions, in line with international regulatory approvals and clinical practice guidelines [9,10]. Nevertheless, several other first- and second-generation antipsychotics were also prescribed, reflecting the substantial degree of off-label use in child and adolescent psychiatry [31]. These real-world prescribing patterns underscore the clinical relevance of observational registry data for understanding both the benefits and risks of antipsychotic treatment outside controlled trial settings [32].

After adjustment for baseline BMI z-score, sex, age, stimulant use, antipsychotic-naïve status, and antipsychotic group, BMI z-scores increased significantly over time across the entire cohort. This finding confirms that weight gain remains a common and clinically relevant adverse effect of antipsychotic treatment in youth [33], regardless of diagnostic category [34]. Baseline BMI z-score was strongly associated with subsequent BMI, indicating that children who begin treatment with higher relative weight are particularly vulnerable to persistent overweight or obesity during follow-up. Together with previous work, these findings suggest that baseline BMI is clinically relevant both as a marker of vulnerability to large relative weight gain and as a predictor of sustained excess weight once treatment has started [35].

We did not detect a statistically significant difference in the average linear rate of BMI z-score increase between children and adolescents with and without ASD. However, the confidence interval of the linear time × ASD interaction was compatible with small to moderate clinically meaningful differences, indicating that our data cannot exclude a modest excess risk in either direction. Importantly, a significant quadratic interaction between time and ASD indicated that the shape of BMI z-score trajectories differed between groups, consistent with a more pronounced early increase followed by an earlier attenuation of weight gain in the ASD group. This finding suggests that diagnostic group differences may relate more to the temporal dynamics of weight gain than to the overall long-term slope. As suggested by Goltz et al. (2019), children with ASD may be affected by SGA differently than children with other psychiatric illnesses [29].

Several mechanisms may underlie this pattern, including heightened sensitivity to antipsychotic-related metabolic effects at younger ages, differences in physical activity or dietary habits, or behavioural features that limit early lifestyle adaptation [36]. The apparent later plateau may reflect treatment adjustments, behavioural interventions, or ceiling effects once higher BMI levels are reached [19]. These explanations remain speculative and cannot be directly tested within the scope of the present registry.

An important consideration in interpreting these findings is the younger age at treatment initiation among participants with ASD. Antipsychotic-associated weight gain is known to be most pronounced in younger children and early in treatment [37,38], and the observed curvature of BMI trajectories may therefore partly reflect developmental timing rather than ASD-specific metabolic vulnerability. Although age was included as a covariate, residual confounding related to pubertal stage and normative growth trajectories cannot be fully excluded.

An additional contextual factor that may have influenced BMI trajectories is the structured follow-up embedded within the SENTIA programme, which includes counselling on diet and physical activity and explicit discussion of metabolic adverse effects [22]. Enhanced monitoring and preventive counselling may have attenuated weight gain over time and reduced between-group differences. This interpretation remains speculative but is consistent with the integrated care model underpinning the registry.

This study has several limitations. Registry-based data derived from routine clinical practice are subject to variability in follow-up intervals and incomplete information for some variables. Prescribing-reason data were available for only a subset of participants. Antipsychotic exposure could only be modelled at baseline by drug class and not as a time-varying variable, and information on dose and cumulative exposure was unavailable. The pubertal stage, a key determinant of BMI trajectories during adolescence, was not assessed. Furthermore, the predominance of boys (particularly within the ASD group) reflects real-world clinical samples but limits the generalisability of our findings to girls and adolescent females and precludes the examination of sex-specific metabolic effects. Although sex was adjusted for in all models, the small number of female participants precluded reliable sex-stratified analyses, and potential influences of pubertal development and hormonal changes could not be assessed. In addition, while longitudinal mixed-effects models accounted for variability in follow-up time, we were unable to formally compare weight trajectories by categorical treatment duration (e.g., short- vs. longer-term exposure), an issue that remains insufficiently addressed in the existing literature [39]. Finally, although the mixed-effects approach accounted for repeated measures and individual heterogeneity, residual confounding and limited statistical power to detect small group differences cannot be excluded.

Despite these limitations, this study provides valuable real-world evidence on antipsychotic prescribing patterns and longitudinal BMI trajectories in a clinically heterogeneous paediatric population. The observation of distinct temporal patterns of weight change in ASD adds nuance to the existing literature and highlights the importance of early preventive strategies. More broadly, this registry demonstrates the potential of systematic post-authorisation monitoring to generate clinically meaningful insights in the context of widespread off-label antipsychotic use in children and adolescents.

## 5. Conclusions

Antipsychotic medications are frequently prescribed to children and adolescents with psychiatric disorders, often in off-label contexts. Youth with ASD tended to initiate antipsychotic treatment at a younger age. Although no robust differences were observed in overall longitudinal BMI z-score trajectories compared with non-ASD peers, the temporal pattern of change differed, with evidence consistent with earlier attenuation of BMI z-score gain in the ASD group. These findings emphasise the need for early and systematic metabolic monitoring in all antipsychotic-treated children and adolescents and highlight the value of registry-based post-authorisation surveillance for informing real-world safety. Collecting systematic safety data in off-licence paediatric prescribing is essential to improve clinical decision-making and optimise preventive strategies.

## Figures and Tables

**Table 1:** Sociodemographic and clinical characteristics of the sample.

Baseline	Total *n* = 266
Age, years—mean (SD) [range]	11.1 (3.6) [3–17.9]
Male sex [*n*, (%)]	204 (76.7)
Ethnicity: Caucasian [*n*, (%)]	205 (91.1)
Main psychiatric diagnosis [*n*, (%)]	
ASD	113 (42.5)
ADHD	97 (36.5)
Conduct disorders ^1^	70 (26.3)
Unspecified neurodevelopmental disorder	17 (6.4)
Depressive disorders	13 (4.9)
Anxiety disorders	11 (4.1)
Tic spectrum disorders ^2^	11 (4.1)
Eating disorders	8 (3.0)
OCD	8 (3.0)
Psychotic disorders	5 (1.9)
Other	8 (3.0)
Substance use [*n*, (%)]	7 (2.6)
Somatic comorbidities [*n*, (%)]	
Neurological disorder	21 (7.9)
Obesity	10 (3.8)
Endocrine disease	8 (3.0)
Hypercholesterolaemia	6 (2.3)
Cardiovascular disease	6 (2.3)
Diabetes	2 (0.8)
Systemic disease	3 (1.1)
Reproductive or sexual disorders	1 (0.4)
Other	109 (41.0)
Antipsychotic medication [*n*, (%)]	
Risperidone	132 (54.1)
Aripiprazole	69 (28.3)
Olanzapine	17 (7.0)
Paliperidone	7 (2.9)
Quetiapine	5 (2.0)
Periciazine	3 (1.2)
Pimozide	3 (1.2)
Tiapride	3 (1.2)
Clozapine	2 (0.8)
Haloperidol	1 (0.4)
Levomepromazine	1 (0.4)
Ziprasidone	1 (0.4)

^1^ Includes unspecified behavioural/conduct disorder, oppositional defiant disorder, and conduct disorder. ^2^ Includes Tourette’s disorder and tic disorder. Percentages of diagnoses are based on the total sample (*n* = 266). Multiple diagnoses per participant were possible. ADHD = attention-deficit/hyperactivity disorder; ASD = autism spectrum disorder; OCD = obsessive–compulsive disorder.

**Table 2:** Family history.

Baseline	Total *n* = 266
Family psychiatric history [*n*, (%)]	
Affective disorders	37 (13.9)
Externalising disorders	27 (10.2)
ASD	25 (9.4)
Anxiety disorders	20 (7.5)
Psychotic disorders	13 (4.9)
Substance use disorders	12 (4.5)
Other	22 (8.3)
Family somatic history [*n*, (%)]	
Diabetes	37 (13.9)
Neurological disorder	29 (10.9)
Cardiovascular disease ^1^	27 (10.2)
Endocrine disease	21 (7.9)
Hypertension	19 (7.1)
Obesity	18 (6.8)
Hypercholesterolaemia	15 (5.6)
Systemic disease	6 (2.3)
Other	76 (28.6)

^1^ Not including hypertension.

**Table 3:** Comparison of individuals with and without ASD.

	ASD *n* = 113	Non-ASD *n* = 153	*p*-Value
Age, years—[mean (SD)]	9.8 (3.9)	12.0 (3.0)	<0.001
Sex, ^†^ male [*n*, (%)]	99 (87.6)	105 (68.6)	<0.001
Under 6 years old [*n*, (%)]	23 (20.4)	3 (2)	<0.001
Psychiatric comorbidity ^1^ [*n*, (%)]	37 (32.7)	46 (30.1)	0.740
Somatic comorbidity ^2^ [*n*, (%)]	62 (54.9)	70 (45.8)	0.178
Family psychiatric history ^3^ [*n*, (%)]	45 (39.8)	59 (38.6)	0.935
Family somatic history ^3^ [*n*, (%)]	54 (47.8)	88 (57.5)	0.148
Antipsychotics naïve [*n*, (%)]	39 (40.6)	59 (43.1)	0.813
Total visits—[mean (SD)]	3.8 (4.2)	4.4 (3.9)	0.055

^1^ Percentage of individuals with more than one psychiatric disorder diagnosis. ^2^ Percentage of individuals with at least one somatic comorbidity. ^3^ Percentage of individuals with at least one family psychiatric or somatic disorder. ^†^ Sex missing in three participants. ASD = autism spectrum disorder; SD = standard deviation.

**Table 4:** BMI categories.

Cut-Off Interpretations for BMI z-Scores ^1^	*n* (%)
Thinness	2 (0.8)
Normal weight	201 (82.0)
Overweight	25 (10.2)
Obesity	17 (6.9)

^1^ Comparison ASD vs. non-ASD (Fisher’s exact test): *p* = 0.542.

**Table 5:** Comparisons of anthropometrics between individuals with and without ASD.

	ASD		Non-ASD		*t* (df)	*p*-Value
	*n*	Mean (SD)	*n*	Mean (SD)		
***z*** **-Height**	102	0.1 (1.2)	142	−0.1 (1.1)	1.15 (209.89)	0.249
***z*** **-Weight**	105	0.2 (1.4)	144	0 (1.1)	1.81 (200.84)	0.071
**BMI**	103	19.3 (4.8)	142	19.8 (4.4)	−0.79 (207.03)	0.429
***z*** **-BMI**	102	0.2 (1.4)	143	0 (1.2)	0.87 (199.28)	0.388

ASD = autism spectrum disorder; SD = standard deviation.

**Table 6:** Linear mixed-effects model for BMI z-scores.

Term	Estimate	SE	df	*t*-Value	*p*-Value
(Intercept)	0.205	0.113	345.3	1.82	0.070
Time (centred)	0.130	0.0536	96.8	2.42	0.017
ASD (vs. non-ASD)	0.0459	0.119	121.3	0.39	0.700
Time^2^ (centred)	−0.0030	0.00574	515.0	−0.52	0.607
Baseline BMI z-score	0.973	0.0151	308.0	64.6	<0.001
Sex (female)	−0.0147	0.0435	300.0	−0.34	0.735
Age	0.00043	0.00541	297.0	0.08	0.937
Stimulant	0.0400	0.0359	262.0	1.11	0.267
AP naïve	0.0548	0.0348	268.0	1.58	0.116
AP group	0.0427	0.0471	277.0	0.91	0.366
Time (centred) × ASD	0.0968	0.0838	96.0	1.16	0.251
Time^2^ (centred) × ASD	−0.0155	0.00756	503.0	−2.05	0.041

Time was centred. Estimates represent fixed effects from a linear mixed-effects model with random intercepts and random slopes for time at the subject level. AP = antipsychotic; ASD = autism spectrum disorder; BMI = body mass index z-score; SE = standard error.

## Data Availability

The data presented in this study are available from the corresponding author upon reasonable request. Access to the data will be subject to an appropriate application and review of the research proposal by the research team, in accordance with ethical, legal, and data protection requirements.
